# A Spanish Consensus on the Use of Safinamide for Parkinson’s Disease in Clinical Practice

**DOI:** 10.3390/brainsci10030176

**Published:** 2020-03-18

**Authors:** Javier Pagonabarraga, José Matías Arbelo, Francisco Grandas, Maria-Rosario Luquin, Pablo Martínez Martín, Mari Cruz Rodríguez-Oroz, Francesc Valldeoriola, Jaime Kulisevsky

**Affiliations:** 1 Movement Disorders Unit, Neurology Department, Hospital de la Santa Creu i Sant Pau, 08041 Barcelona, Spain; jkulisevsky@santpau.cat; 2 Department of Medicine, Autonomous University of Barcelona, 08193 Barcelona, Spain; 3 Centro de Investigación en Red sobre Enfermedades Neurodegenerativas (CIBERNED), 28031 Madrid, Spain; 4 Movement Disorders Unit, Neurology Department, Hospital Universitario San Roque, 35001 Las Palmas, Spain; jmarbelo@gmail.com; 5 Department of Medicine, Universidad Fernando Pessoa-Canarias, 35450 Las Palmas, Spain; 6 Movement Disorders Unit-CSUR, Neurology Department, Hospital General Universitario Gregorio Marañón, 28007 Madrid, Spain; francisco.grandas@salud.madrid.org; 7 Department of Medicine, Universidad Complutense de Madrid, 28040 Madrid, Spain; 8 Movement Disorders Unit, Clínica Universidad de Navarra (CUN), 31008 Pamplona, Spain; rluquin@unav.es; 9 Navarra Institute for Health Research (IdiSNA), 31008 Pamplona, Spain; 10 Instituto de Salud Carlos III, 28029 Madrid, Spain; pmm650@hotmail.com; 11 Centro de Investigación Biomédica en Red sobre Enfermedades Neurodegenerativas (CIBERNED), 28031 Madrid, Spain; 12 Neurology and Neuroscience Unit, Clínica Universidad de Navarra (CUN), 31008Pamplona, Spain; mcroroz@unav.es; 13 Centre for Applied Medical Research (CIMA), 31008 Pamplona, Spain; 14 Neurosciences Institut, Hospital Clinic de Barcelona, 08036 Barcelona, Spain; FVALLDE@clinic.ub.es; 15 Department of Medicine, Universitat de Barcelona, 08036 Barcelona, Spain; 16 Biomedical Research Institute (IIB-Sant Pau), 08041 Barcelona, Spain; 17 Department of Medicine, Universitat Oberta de Catalunya, 08018 Barcelona, Spain

**Keywords:** safinamide, efficacy, safety, fluctuations, dyskinesia, RAND/UCLA appropriateness method

## Abstract

Safinamide is an approved drug for the treatment of motor fluctuations in Parkinson’s disease (PD). Scarce data are available on its use in clinical practice. A group of Spanish movement disorders specialists was convened to review the use of safinamide across different clinical scenarios that may guide neurologists in clinical practice. Eight specialists with recognized expertise in PD management elaborated the statements based on available evidence in the literature and on their clinical experience. The RAND/UCLA method was carried, with final conclusions accepted after a 2-round modified Delphi process. Higher level of agreement between panellists was reached for the following statements. Safinamide significantly improves mean daily OFF time without troublesome dyskinesias. Adjunctive treatment with safinamide is associated with motor improvements in patients with mid-to-late PD. The efficacy of safinamide on motor fluctuations is maintained at long-term, with no increase over time in dyskinesias severity. The clinical benefits of safinamide on pain and depression remain unclear. Safinamide presents a similar incidence of adverse events compared with placebo. The efficacy and safety of safinamide shown in the pivotal clinical trials are reproduced in clinical practice, with improvement of parkinsonian symptoms, decrease of daily OFF time, control of dyskinesias at the long term, and good tolerability and safety.

## 1. Introduction

The management of Parkinson’s disease (PD) has improved over the last 20 years with the development of different families of dopaminergic drugs with specific mechanisms of action. Dopamine agonists (DA), in monotherapy or combination with levodopa, are associated with a reduction and delay in the development of motor fluctuations and dyskinesias [1].

When, despite the use of dopaminergic drugs, patients develop motor fluctuations and/or dyskinesias, treatment with Monoamine oxidase B (MAO-B) inhibitors (safinamide, rasagiline, or selegiline), Catechol-O-Methyl-Transferase (COMT) inhibitors (opicapone, entacapone, or tolcapone), subcutaneous apomorphine, amantadine, and/or zonisamide is recommended [2]. 

Safinamide (Xadago®, Zambon S.p.A) is an α-aminoamide derivative with a dual mechanism of action targeting dopaminergic and non-dopaminergic pathways. Besides the selective inhibition of MAO-B, safinamide can modulate glutamate release via sodium channel blockade and calcium channel modulation [3]. This dual mechanism of action results in the increased availability of dopamine within the striatum and the decreased release of glutamate in regions with glutamatergic hyperexcitability [4]. Glutamate hyperexcitability restricted to cortical motor areas and functionally related regions of basal ganglia develop early in untreated PD patients. Additionally, postsynaptic striatal changes in the density and sensitization of glutamatergic receptors are crucial for the development of fluctuations and dyskinesias [5].

The efficacy and safety of safinamide were demonstrated in different pivotal, multicentric, double-blind, placebo-controlled clinical trials, which granted its marketing authorization [6,7,8]. Safinamide was approved in 2015 in the EU and in 2017 in the US as adjunctive therapy to levodopa alone or in combination with other PD medicinal products in mid- to late-stage fluctuating patients [9,10]. 

These studies included patients with early [11,12] and advanced PD [6,7,8,13], showing the short- and long-term benefit of safinamide for motor and non-motor symptoms. Unlike other MAO-B inhibitors, the inhibition of safinamide is reversible and more selective compared with that of selegiline and rasagiline, minimizing the risk of hypertensive crises or serotonergic syndrome, and preventing dietary restrictions [14]. Safinamide is contraindicated in combination with other MAO-B inhibitors, such as rasagiline, but it can be prescribed to patients receiving amantadine, since they modulate the glutamatergic system through different mechanisms of action [10].

Because safinamide was recently commercialized, there are limited data to date on its use in routine clinical practice. A group of Spanish movement disorders specialists was therefore convened to review the use of safinamide across different clinical scenarios and guide neurologists assisting PD patients in clinical practice. This process culminated with the development of a comprehensive set of statements based on the evidence available in the literature and the clinical experience of the experts. 

## 2. Materials and Methods

### 2.1. Expert Panel Composition

The panel of experts was composed of 8 movement disorders specialists with recognized expertise in PD management within the Spanish National Health System and with proven research experience. The panellists elaborated the statements in light of the evidence available in the literature and based on their clinical practice experience. The RAND/UCLA method [15] was carried out with the assistance of experienced methodologists. 

### 2.2. The RAND/UCLA Method

We used the RAND/UCLA method [15] to develop a set of statements (recommendations and conclusions) and rate the level of consensus achieved. In brief, the expert panel developed a group of questions related to the use of safinamide across five clinical scenarios: (1) efficacy of safinamide and indications of use, (2) adverse events associated with safinamide, (3) clinical profile of patients that may benefit from safinamide treatment in clinical practice, (4) treatment switch from rasagiline to safinamide, and (5) concomitant use of safinamide and antidepressants. Then, the latest available literature was reviewed and synthesized. Based on available evidence and their judgement, each expert answered the questions, and the answers were summarized as conclusions and recommendations. The panellists finally rated each statement in a 2-round modified Delphi process. 

### 2.3. Literature Search Strategy

Systematic literature searches were conducted in February 2018 for Spanish- and English-written articles related to safinamide treatment using the following databases: Pubmed, Clinicaltrials.gov, Google Scholar, European Neurological Review, and the Cochrane database. Abstracts on safinamide treatment were also retrieved from PD-related congresses. Additionally, the Spanish Agency for Medicines and Health Products (AEMPs) was also searched for entries associated with safinamide treatment. The literature search approach was conducted in two phases: (1) general search using the keyword “safinamide”, and (2) specific search including publications on MAO-B and COMT inhibitors. Specific keywords were used in this latter phase depending on the clinical scenario. 

### 2.4. Delphi Rounds

The panellists rated each statement on a 4-point Likert scale (1 = totally disagree, 4 = totally agree) in a 2-round modified Delphi process. Additionally, they provided feedback for the refinement of items when appropriate. 

Consensus was established when ≥80% of experts agreed with the statement. Those statements with a level of agreement <80% were discussed and refined, if necessary, during the on-site meeting. Each statement was classified by the Level of Evidence (LE) and Grade of Recommendation (RG) according to the recommendations of the Oxford Centre for Evidence-based Medicine [16]. 

During the first round, panellists rated each statement anonymously without the interaction with other panellists. The facilitator integrated first-round responses, which were used in the on-site meeting to allow for re-rating scores or rephrasing items. The statements were finally modified during the second Delphi round considering the insights from the expert panel. The statements presented in this study are the result of the second Delphi round. 

## 3. Results

### 3.1. Efficacy

A large number of studies provided evidence of the efficacy of safinamide at 50 and 100 mg/day in the early and mid-to-late PD stages, showing significant benefits in motor and non-motor symptoms. The main efficacy outcomes reported in pivotal trials of safinamide in PD are summarized in Table 1. 

#### 3.1.1. De Novo

Although its mechanism of action and preliminary results in animals may suggest a neuroprotective function of safinamide, there are not yet studies conducted in humans confirming this effect [3,17,18]. 

The impact of safinamide in de novo patients deserves more investigation [18,19,20], as only one study investigated safinamide response in non-treated (de novo) patients and in patients under a stable treatment with DA, but results of de novo subgroup are not detailed in the study [21].

- *Conclusion: There is no current evidence on the benefit of safinamide as de novo or neuroprotector treatment, or to treat hypersomnia. Level of agreement: 88%. LE: 5.*

#### 3.1.2. Motor Symptoms

The benefits of safinamide in improving motor function, fluctuations and dyskinesias have been demonstrated in different randomized controlled trials (RCTs) and observational studies. 

##### Motor function

Motor function improved in patients with early PD receiving safinamide and a stable dose of a single DA, as measured with the Unified Parkinson’s Disease Rating Scale (UPDRS) part III total score [11,12]. The exploratory analysis of Study 015 showed significantly greater improvements in the UPDRS-III in patients receiving safinamide 100 mg/day compared with those receiving the placebo (−6.0 vs. −3.6; *P* = 0.0419), whereas the safinamide 200 mg/day group did not differ significantly from the placebo group [11]. The improvement in motor function was sustained in the 12-month extension study (Study 017) [12]. 

Two phase III RCTs and one retrospective study support the beneficial effect in motor scales of safinamide as levodopa adjunct in mid- to late-stage fluctuating patients [6,7,8,13]. Study 016 showed significant improvements in the UPDRS-III with 50 and 100 mg/day safinamide dosages compared with the placebo (difference vs. placebo: −1.8 and −2.6, respectively) [6]. Similar results were observed in the 2-year extension period (Study 018), reporting long-term improvements in UPDRS-II, -III, and -IV in the safinamide 100 mg/day group [7]. In the Settle study, safinamide 100 mg/day significantly decreased (improved) the UPDRS-III score from baseline to Week 24 compared with the placebo (difference vs. placebo: −1.8; *P* = 0.003) [8]. In line with these results, the retrospective study of Mancini et al. reported a reduction in motor scales after the introduction of safinamide treatment [13].

- *Conclusion: Clinical trials and observational studies demonstrated that adjunctive treatment with safinamide is associated with motor improvements in patients with mid-to-late PD and motor deficit (treated with levodopa alone or in combination with PD treatment(s))* [6,7,8,13]. *Level of agreement: 100%. LE: 2.*

##### Fluctuations

The efficacy of safinamide in the control of motor fluctuations was described in 3 RCTs, including 1762 patients. In Study 016, treatment with 50 and 100 mg/day safinamide significantly increased ON time without or with non-troublesome dyskinesias by 0.51 (*P* = 0.0223) and 0.55 h (*P* = 0.0130), respectively, compared with the placebo [6]. Differences between the 100 mg/day safinamide and placebo groups remained significant after 18 months in the extension study [7]. In the Settle study, ON time without troublesome dyskinesia was increased by 0.96 h in the safinamide group compared with the placebo group (*P* < 0.001) [8]. Significant differences were observed between the safinamide and placebo groups in the OFF time reduction at months 6 [6,8] and 24 [7]. 

In the pooled analysis of data from studies 016 and Settle, safinamide resulted in a significant improvement in mean daily ON time without or with non-troublesome dyskinesias and in OFF time, regardless of the concomitant treatment with DA, COMT inhibitors, and amantadine [22]. 

Positive results from RCTs are consistent with clinical practice studies. In the retrospective study of Mancini et al., patients treated with 50 mg/day safinamide significantly improved the time spent in OFF and in ON with dyskinesias, while those receiving 100 mg/day only achieved significant differences for the time in OFF. These different results could be explained because a minority of patients in this sample received 100 mg/day of safinamide (24%), and because time spent in OFF at baseline was significantly longer in the group receiving 100 mg/day (90 min, first quartile; third quartile 60;120) than in the 50 mg/day group (60 min, first quartile; third quartile 60;72.5) (*P* < 0.0014) [13]. In the prospective observational study of Pagonabarraga et al., safinamide was associated with an improvement in typical parkinsonism symptoms during the wearing-off, with a mean OFF time reduction of 0.9 h/day after 3 months of treatment (*P* < 0.001) [23].

- *Conclusion: Safinamide treatment is efficacious in patients with motor fluctuations* [6,7,8,13,23,24,25]. *Level of agreement: 100%. LE: 1.*

##### Dyskinesias

In Study 016, the Dyskinesia Rating Scale (DRS) score during ON time was comparable between the safinamide and placebo groups, reflecting no increased severity of dyskinesias with safinamide [6]. In the 018-extension study, no significant differences were observed between active and comparator groups in the change of DRS scores despite the 31% and 27% reduction with safinamide 50 and 100 mg/day, respectively, vs. the 3% observed with the placebo. However, the authors explain this result by the small proportion of patients with moderate-to-severe dyskinesia at baseline, which is further supported by the significant improvement in dyskinesias observed with safinamide 100 mg/day in the subset of patients with moderate-to-severe dyskinesia (difference vs. placebo: −1.50; *P* = 0.0317) [7]. Likewise, the Settle study revealed comparable changes in DRS scores from baseline to Week 24 in the safinamide and placebo groups [8]. 

In the post-hoc analysis of Study 018, a lower proportion of patients presented worse DRS in both the safinamide groups compared with the placebo, regardless of the change in levodopa dose [25].

- *Conclusion: No clear conclusion regarding the direct effect of Safinamide on dyskinesias was achieved.*

#### 3.1.3. Non-Motor Symptoms

The mechanism of action of safinamide may imply a potential beneficial effect on non-motor symptoms, including pain or depression. 

##### Pain

A pooled analysis of data from Study 016 [6] and Settle [8] showed a significant reduction in the number of patients receiving concomitant pain treatments in the safinamide group compared with the placebo group (reduction vs. placebo: 23.6%; *P* = 0.0421) [26]. Safinamide 100 mg/day significantly improved 2 out of 3 pain-related items of the Parkinson’s Disease Quality of Life Questionnaire-39 (PDQ-39) of the bodily discomfort domain. Although this exploratory analysis presents some limitations, the results showed the beneficial effect of safinamide 100 mg/day on pain, with 79.7% of the improvement being directly attributed to the intrinsic effect of safinamide [26]. A retrospective study has recently shown that patients treated with safinamide significantly reduced the pain item of the Parkinson’s Disease Questionnaire-8 (PDQ-8), but there are no published studies showing a significant improvement by safinamide in specific pain scales [27].

##### Depression and cognitive impairment

In patients with early PD (Study 015), differences between the safinamide and placebo groups in the change from baseline to Week 24 for the Hamilton Rating Scale for Depression (HAM-D) and the Mini-Mental State Examination (MMSE) were not significant (although data are not shown for these variables) [11]. 

Heterogeneous results were observed in mid-to-late PD patients. In Study 016, a non-significantly greater reduction in the GRID Hamilton Rating Scale for Depression (GRID-HAM-D) score from baseline to Week 24 was observed in the safinamide group compared with the placebo group [6]. Differences reached statistical significance in the 18-month extension study, although data are not shown for the GRID-HAM-D [7]. In the Settle study, the safinamide group failed to achieve a significant change from baseline to Week 24 in GRID-HAM-D and MMSE [8]. 

The pooled analysis of studies 016 and 018 showed a significant improvement in GRID-HAM-D in the safinamide 100 mg/day group vs placebo (mean difference vs. placebo: −0.57; *P* = 0.0408) that was maintained after 24 months of treatment (mean difference vs. placebo: −0.87; *P* = 0.0027). In line with these results, the “Emotional well-being” domain of the PDQ-39 was better perceived in the safinamide group after 6 (mean difference: −3.77; *P* = 0.0067) and 24 months (mean difference: −4.66; *P* = 0.0006), with a lower proportion of patients reporting depression in this group [28].

- *Conclusion: In patients with PD, the clinical benefits of safinamide on pain, depression, or cognitive impairment remain unclear, as available evidence is indirect and inconsistent* [7,8,26]. *Level of agreement: 100%. LE: 3.*

##### Sleep disturbances 

A retrospective analysis compared the interference of safinamide and rasagiline on sleep disturbances and daytime sleepiness in patients with fluctuating PD via the Parkinson’s Disease Sleep Scale 2 (PDSS2), Pittsburgh Sleep Quality Index (PSQI), and Epworth Sleepiness Scale (ESS). PDSS2 and ESS scores significantly improved in safinamide recipients (*n* = 46), contrasting with rasagiline-treated patients (*n* = 15) who did not show significant differences [29]. Interestingly, these authors also showed that, in two motor fluctuating PD cases, safinamide 100 mg/day added to levodopa was effective at improving restless legs syndrome and frequent periodic limb movements [30]. Furthermore, the recently published retrospective study of Bianchi et al. showed a significant reduction in the “sleep/fatigue” domain associated with safinamide [27].

- *Conclusion: No clear conclusion regarding the clinical benefits of safinamide on sleep disturbances was achieved.*

### 3.2. Safety 

Safinamide was well tolerated in different RCTs and observational studies, with a similar incidence of treatment-emergent adverse events (TEAEs) compared with the placebo, most of them being of mild-to-moderate severity. The main safety outcomes reported in pivotal trials of safinamide in PD are summarized in Table 1.

#### 3.2.1. Most Common AEs

In patients with early PD (Studies 015 and 017), a low and comparable incidence of TEAEs between the safinamide and placebo groups was observed. Most of the TEAEs were mild or moderate in severity, and none of the serious TEAEs was considered related to the treatment [11,12]. 

In mid-to-late PD patients, short- and long-term therapy with safinamide was characterized by a low incidence of TEAEs [6,7,8]. Studies 016, 018, and Settle reported a comparable incidence of TEAEs and TEAEs, leading to discontinuations between the safinamide and placebo groups [6,7,8]. Moreover, only dyskinesia occurred more frequently in the safinamide groups compared with the placebo group [6,7,8]. In Study 016, a higher proportion of patients reported worsening of PD and depression in the placebo group vs. safinamide groups, and a higher frequency of serious AEs (SAEs) was observed in the placebo (8.1%) and safinamide 100 mg/day (9.8%) groups compared with the safinamide 50 mg/day group (3.6%) [6]. In the Settle study, fewer SAEs were observed in the safinamide group vs. the placebo group (6.6% vs. 9.5%, respectively) [8]. 

Combined data from the safety population of Studies 016 and 018 supported the good tolerability of safinamide in the long term, with a similar rate of serious TEAEs among groups and a lack of specific AEs [7]. 

In the RCT conducted by Marquet et al., safinamide administered at therapeutic (100 mg) or supratherapeutic (350 mg) doses did not result in a clinically relevant increase in the pressor effect of oral tyramine, suggesting that safinamide can be administered without any dietary restrictions related to tyramine-containing foods [14]. 

Observational studies also investigated the tolerability of safinamide, showing no specific safety issues. In the prospective study of Pagonabarraga, 15 (31.2%) safinamide recipients experienced AEs, 9 of which were of mild severity. A total of six (12.7%) patients (all of them ≥75 years) discontinued safinamide due to the development of confusional syndrome, three treated with 50 mg/day and three with 100 mg/day. Among them, two patients previously developed confusional syndrome when treated with amantadine [23]. 

In a published Spanish consensus on the use of safinamide, the experts reported the lack of SAEs associated with safinamide in clinical practice and described individual cases of headache, sleepiness, general discomfort, confusion, nervousness, and epigastralgia [24]. This consensus was based on local meetings between neurologists assisting PD patients with more than one year of experience with safinamide. Final consensus recommendations were based on common observations on the benefits of safinamide in clinical practice. 

A meta-analysis comparing the efficacy and safety of safinamide and entacapone as add-on treatment to levodopa in fluctuating PD patients showed no significant differences in study discontinuations due to AEs and deaths, although they were numerically lower in the safinamide group [31].

- *Conclusion: Most frequent AEs (≥5% incidence) associated with safinamide treatment are scotoma, blurred vision, dizziness, abdominal and lumbar pain, nausea, and hypertension; they occur in a similar frequency than that observed in patients treated with placebo* [6,7,8,11,12,14,23,24,31]. *Level of agreement: 100%. LE: 1.*
- *Conclusion: There is a higher risk of confusional syndrome with the 100 mg dose in elderly patients (≥75 years) with clinically relevant cognitive dysfunction, or in more advanced PD stages (H&Y>3)* [6,7]. *Level of agreement: 88%. LE: 1.*

#### 3.2.2. Dyskinesias

In patients with PD and motor fluctuations, treatment with safinamide as adjunctive therapy to levodopa increases dopamine levels, which could lead to the emergence or worsening of dyskinesias. The rates of severe dyskinesia in Study 016 were 0.8% in the safinamide 100 mg/day group, 0.9% in the 50 mg/day group, and 2.3% in the placebo group [6]. The Settle study reported a rate of severe dyskinesia of 1.8% in patients receiving safinamide and of 0.4% in patients receiving placebo [8]. In Study 018, the authors described a comparable incidence of new/worsening dyskinesias in the safinamide and placebo groups [7]. 

In light of this evidence, different approaches are proposed to prevent the emergence of worsening of dyskinesias [23,32,33]. 

##### Prevention of de novo dyskinesias 

The observational study of Pagonabarraga et al. suggests initiating treatment with safinamide 100 mg/day in patients with previously reported troublesome dyskinesias to avoid new dyskinesias [23]. 

##### Prevention of worsening of pre-existing dyskinesias

Two approaches have been suggested to prevent worsening of pre-existing dyskinesias: (1) to reduce levodopa dose or (2) to initiate treatment with 100 mg/day safinamide. The investigators of two reviews advocate for a reduction of levodopa dose in clinical practice when adding safinamide to an anti-parkinsonian regimen [33,34]. To avoid disabling dyskinesias, the observational study of Pagonabarraga et al. suggests initiating treatment with safinamide 100 mg/day when dyskinesias are clinically burdensome [23]. The study of Borgohain et al. draw similar conclusions, given that patients with moderate-severe dyskinesias (DRS >4) improved with safinamide 100 mg [7]. 

##### Treatment of developing or worsening dyskinesias 

According to the panel of experts of this study, different approaches may be undertaken in patients with motor fluctuations who develop or worsen pre-existing dyskinesias after treatment with safinamide: (1) reduction of levodopa dose, (2) increase of safinamide dosage to 100 mg/day, or (3) introduction of amantadine treatment. 

The reduction of levodopa dose strategy is supported by three reviews and one observational prospective study. In the review carried out by deSouza et al., the authors propose to reduce the levodopa dose or to introduce safinamide, an MAO-B inhibitor, or a COMT inhibitor [32]. Similarly, Müller et al. recommend the cautious reduction of oral levodopa following the addition of safinamide to prevent that the substituting properties of safinamide may aggravate dyskinesias [34]. In agreement with these recommendations, the observational study of Pagonabarraga et al. showed an improvement in dyskinesias after increasing the dosage of safinamide to 100 mg/day (three patients) and decreasing levodopa dose (four patients) [23]. 

The increase of safinamide dosage from 50 to 100 mg/day is indirectly backed by Study 018, showing that 33% of patients with moderate-severe dyskinesias improved with safinamide 100 mg/day [7]. As previously indicated, Pagonabarraga et al. registered three patients that resolved new dyskinesias after increasing safinamide dosage to 100 mg/day [23]. 

Alternatively, according to an expert review, the introduction of amantadine can be considered if dyskinesias become troublesome after levodopa reduction [32].

- *Recommendation: In patients with PD and pre-existing troublesome dyskinesias before the introduction of safinamide, the reduction of levodopa dose and the initiation of safinamide treatment at 100 mg/day could be considered* [7,23]. *Level of agreement: 100%. LE: 3; RG: C.*
- *Recommendation: In patients with PD and new or worsening dyskinesias after safinamide treatment, the reduction of levodopa dose or the increase of safinamide dose to 100 mg could be considered* [23,32,33,34]. *Level of agreement: 100%. LE: 5. RG: D.*

#### 3.2.3. Confusional Syndrome

There is interest in studying the impact of safinamide on confusional syndrome, as dopaminergic medications are often associated with neuropsychiatric symptoms or confusional syndrome [35]. 

One case of a patient who developed hypersexuality after five months of treatment with safinamide as add-on therapy for PD has been reported. Although it is unclear why hypersexuality appeared five months after the switch from rasagiline to safinamide, the fact that it disappeared after safinamide withdrawal may suggest the association of safinamide and hypersexuality in this patient [36]. 

In the observational study of Pagonabarraga et al., 12.6% of patients developed confusional syndrome manifested as somnolence, psychomotor agitation, and visual hallucinations that disappeared after safinamide withdrawal. Importantly, these manifestations were observed with both 50 and 100 mg/day safinamide dosages, and in older patients with advanced PD. Two of them previously presented confusional syndrome with amantadine [23].

- *Conclusion: In patients with PD receiving safinamide, the emergence of episodes of confusion, visual hallucinations and somnolence could be possible with 50 and 100 mg doses, and they could disappear after safinamide withdrawal* [23]. *However, there is not enough data to support this association. Leve lof agreement: 88%. LE: 3.*
- *Conclusion: There might be an increased risk for episodes of confusion in patients treated with safinamide with previous documentation of these complications, older age, and advanced PD* [23]. *Level of agreement: 100%. LE: 3.*

### 3.3. Patient Profiles 

In light of efficacy and safety data from RCTs and observational studies and their own experience, the expert panel of this study identified different patient profiles that may benefit from safinamide treatment in clinical practice. 

#### 3.3.1. Without Motor Fluctuations

Three RCTs support safinamide treatment in patients without motor fluctuations, showing short- and long-term improvements in motor scales with no reported SAEs and delayed median time to intervention [11,12]. Study 009 showed a higher proportion of responders in the safinamide groups compared with the placebo group [21]. Similarly, the consensus document from Spanish experts considered that safinamide might be efficacious in patients with PD without fluctuations and uncontrolled parkinsonian symptoms [24]. 

There is no specific evidence supporting the use of safinamide in patients with nocturnal parkinsonian symptoms without evident diurnal fluctuations; neither in patients with morning bradykinesia without evident diurnal fluctuations.

- *Conclusion: Safinamide treatment is efficacious in patients with PD without motor fluctuations receiving agonists and/or levodopa and requiring higher dopaminergic stimulation* [11,12,21,24]. *Level of agreement: 100%. LE: 1.*

#### 3.3.2. With Motor Fluctuations

Extensive evidence exists on the efficacy of safinamide in mid- to late-stage fluctuating patients, which granted its authorization for this subgroup of patients. Safinamide improved motor function with no worsening of dyskinesias, providing an early and sustained response [6,7,8]. 

Observational studies confirm the effectiveness and safety of safinamide for the control of motor fluctuations in patients with dyskinesias [13,23]. In the recently published Spanish consensus, the experts identified that patients with motor fluctuations, particularly of mild-to-moderate severity, experience greater improvements [24].

- *Conclusion: Safinamide treatment is efficacious in patients with motor fluctuations* [6,7,8,13,23,24,25]. *Level of agreement: 100%. LE: 1.*

#### 3.3.3. With Non-Motor Fluctuations

##### Pain 

The effects of safinamide on pain management were evidenced in two post-hoc analyses. These studies showed a 23.6% reduction in the number of patients receiving concomitant pain treatments in the safinamide 100 mg/day group compared with the placebo group, together with an improvement in the PDQ-39 “Bodily discomfort” [26] and the persistence of analgesic effects over two years [37]. 

##### Depression and cognitive impairment

Patients with mid-to-late PD receiving safinamide (50 or 100 mg/day) significantly improved the GRID-HAM-D, PDQ-39 “Emotional well-being”, and the incidence of depression decreased [28]. Results failed to achieve statistical significance in patients with early-stage PD [11].

- *Conclusion: Safinamide treatment could be beneficial in patients with motor fluctuations and depressive symptoms or pain* [26,28,37]. *Level of agreement: 100%. LE: 2.*

##### Sleep disturbances 

Evidence in this subgroup of patients is based on a retrospective analysis of data showing a significant improvement in sleep-related scales in safinamide recipients [29]. In two patients with PD and motor fluctuations, an alleviation of restless legs syndrome and frequent periodic limb movements in sleep was observed [30]. 

#### 3.3.4. Other Profiles

No previous study supports the use of safinamide in patients with Impulse Control Disorders (ICDs) or at risk for developing ICDs, although in the recently published Spanish consensus some experts indicated the potential interest of safinamide for this subgroup of patients [24]. 

There is limited evidence in patients with bilateral deep brain stimulation of the subthalamic nucleus, in those receiving treatment with levodopa/carbidopa, or those receiving treatment with an apomorphine pump. 

### 3.4. Switch from Rasagiline to Safinamide

Mancini et al. reported that patients switching from an MAO-B inhibitor to safinamide significantly improved time in OFF and decreased levodopa and levodopa equivalent dose (LEDD) [13]. Similar conclusions were achieved by the Spanish consensus on the use of safinamide [24]. 

In the prospective study of Pagonabarraga et al., switching from rasagiline to safinamide was associated with a moderate improvement in “wearing-off” and parkinsonism (two patients), decrease in biphasic dyskinesias (one patient), and generalized choreoathetosis (two patients) [23]. 

Martí et al. recently conducted a retrospective-prospective cohort study on 213 PD patients who started safinamide treatment (54% previously on rasagiline), showing an improvement in motor function without worsening of dyskinesias. The incidence of AEs was not significantly different between patients previously treated with rasagiline and those starting directly with safinamide [38,39]. 

Despite some evidence, there is no standardized procedure for the switch from rasagiline to safinamide as only three observational studies and one expert consensus have addressed this issue before [13,23,24,38]. 

In clinical practice studies, MAO-B inhibitors were withdrawn two weeks before starting safinamide treatment [13,23]. In the expert consensus, some neurologists recommend a washout period of two weeks (according to licensing recommendations), others advocate for a shorter washout period, and others to start safinamide treatment immediately after rasagiline withdrawal [24]. In the retrospective study of Martí et al., patients underwent an overnight switch from rasagiline to safinamide with no significant AEs [38]. Regarding the starting dosage of safinamide after rasagiline withdrawal, two strategies were suggested: (1) a starting dosage of 50 mg/day with the potential increase to 100 mg/day when no AEs develop and efficacy is not achieved, or (2) a starting dosage of 100 mg/day since previously published studies did not report significant differences in terms of AEs between these two dosages [6,25].

- *Conclusion: In patients with PD, signs and symptoms that could lead to a switch from rasagiline to safinamide are uncontrolled motor complications, including motor fluctuations and dyskinesias* [13,24]. *Level of agreement: 100%. LE: 4.*
- *Conclusion: The switch from rasagiline to safinamide associated with levodopa could result, in certain situations, in an improvement in motor status and even in non-motor symptoms* [13,23,39]. *Level of agreement: 100%. LE: 5.*
- *Conclusion: In patients with PD previously treated with rasagiline for whom a switch to safinamide is advisable, the 2-week washout period could be avoided and safinamide could be started from one day to another, although there is not enough evidence to support this conclusion* [24]. *Level of agreement: 100%. LE: 5.*
- *Recommendation: In patients with PD previously treated with rasagiline for whom a switch to safinamide is advisable, a starting safinamide dosage of 50 or 100 mg/day could be recommended as in other scenarios. Level of agreement: 100%. LE: 5. RG: D* [24].

### 3.5. Concomitant Use of Safinamide and Antidepressants

Since antidepressants and anxiolytics are commonly prescribed in patients with PD due to the coexistence of depressive symptoms in these patients, there is concern that the potential interaction of both medicinal products could cause serotonin syndrome. 

According to the summary of product characteristics, a washout period of five half-lives of the selective serotonin reuptake inhibitors (SSRI) used previously should be considered before safinamide treatment initiation. The special precautions for use also indicate to avoid the concomitant use of safinamide and fluvoxamine, and when the concurrent treatment is necessary, to use them at the lowest effective dose [10]. 

However, it is important to stress that although these recommendations have a theoretical rationale, the interaction between safinamide and antidepressants has not been demonstrated. 

There are some individual cases reporting serotonin syndrome arising from a possible interaction between SSRI and MAO-B inhibitors, such as one clinical case in which a woman was concomitantly treated with selegiline and nortriptyline [40]. 

A recent literature review was conducted to investigate the potential interaction between MAO-B inhibitors and SSRIs in patients with PD [41]. Based on the literature evidence [42,43], the authors concluded that SSRIs and MAO-B inhibitors could be administered concomitantly if they do not exceed the recommended doses and the SSRI is administered at the lower end of the therapeutic range [41]. 

However, there is no specific study assessing the effects of the coadministration of safinamide and antidepressants. Clinical studies allowing the use of SSRIs did not report additional complications associated with the concomitant use of these drugs [6,7,8]. The published Spanish consensus considered the combination of safinamide and SSRIs, serotonin–norepinephrine reuptake inhibitors, and tricyclic antidepressants as safe, claiming special attention with the use of fluvoxamine and safinamide, and with those receiving high doses of antidepressants [24]. 

A review carried out in 2015 determined that combination therapy (MAO and antidepressants or stimulants) may be considered for the management of treatment-resistant depression (TRD) in non-responders to monotherapy or to other combinations of antidepressants, provided that they are closely monitored [44].

- *Conclusion: Although there is a rational basis on the negative interaction between safinamide and antidepressants, there is not enough evidence supporting this effect to date* [24,41,44]. *Level of agreement: 100%. LE: 5.*
- *Recommendation: The concomitant use of safinamide and antidepressants could be prescribed in strictly necessary cases, with caution, and at the lowest therapeutic dosage* [24,41]. *Level of agreement: 100%. LE: 5; RG: D.*

## 4. Discussion

The unique mechanism of action of safinamide may offer multiple treatment possibilities. However, most of the studies have focused on the impact of safinamide in mid-to-late fluctuating PD patients. Safinamide is a quite young drug, it is not present yet in many countries, and some neurologist may feel unsure about its use in clinical practice. This comprehensive review of clinical trials and studies describing the clinical benefits of safinamide in the real world can be clinically relevant for a large proportion of neurologists. A panel of Spanish experts was convened with the aim of providing treatment guidance across different clinical scenarios. To this end, the latest available evidence was summarized and integrated together with the extensive clinical experience of the panel of experts. The outcome is a comprehensive set of statements on the use of safinamide in clinical practice that are classified by their level of evidence. 

When new drugs are available for the management of PD, it is important to consider the disease symptoms that can be better controlled, the complications that can be diminished by its use, and the potential adverse events and drug interactions that may have a negative impact in the disease. Patients with PD are particularly at risk of adverse drug events since they are typically treated by polypharmacy (i.e., five or more different types of medication) [45]. Polypharmacotherapy includes significant risks as a result of potential interactions between antiparkinsonian drugs and other CNS-active or QT-prolonging drugs [46]. Dopamine agonists have been related to severe neuropsychiatric complications (impulse control disorders, psychosis) whose severity depends partly on dose levels. Amantadine use in PD is related to a higher risk of severe cardiac arrhythmias when combined with antipsychotics and tri- and tetracyclic antidepressants, it increases pramipexole blood levels and CNS toxicity because of common renal excretion, and its anticholinergic effects may impair cognitive function and trigger psychotic episodes in elderly patients [47]. 

In this scenario, safinamide, with its higher selectivity for inhibiting MAO-B compared with rasagiline, has a safer profile regarding the potential development of serotonergic syndrome and hypertensive crisis. In addition, safinamide is not metabolized by CYP1A2 so, unlike rasagiline, its plasma concentrations are not modified by commonly used drugs or substances that are metabolized by this cytochrome (fluoroquinolone, verapamil, amiodarone, insulin, modafinil, omeprazole, carbamazepine, valproate, caffeine, tobacco, broccoli, etc.). On the other hand, safinamide can inhibit the breast cancer resistance protein, although no studies have analyzed this interaction clinically, and it is not known whether this interaction may cause relevant changes in plasma concentrations of some chemotherapeutic agents, prazosin, teriflunomide, chlorothiazide, pantoprazole, or some statins. 

Published observational retrospective studies have shown the ability of safinamide to manage PD symptoms using lower doses of both L-Dopa and dopamine agonists, which may decrease motor and non-motor complications in the mid and long term, and its indirect effect on the glutamatergic system also appears safer compared with the specific antagonistic NMDA effect of amantadine. Only in elderly patients (>75 years) with clinically relevant cognitive dysfunction the use of safinamide seems to increase the development of confusional syndrome. No other explicit limitations account for the use of safinamide in geriatric PD patients with polypharmacotherapy.

This report covers both clinical scenarios with extensive clinical evidence and those yet unexplored that require further investigation. Importantly, the recommendations and conclusions provided cannot be understood as categorical assertions but rather as a guide for specialists. A significant limitation is that the panel of experts was only composed of Spanish specialists, and specific aspects may thus not be generalizable to other countries. 

The RAND/UCLA method was used to obtain consensus on the statements. This methodology is a well-established approach and particularly useful for clinical fields with scarce evidence [15]. Remarkably, a high level of agreement was achieved for most of the statements, even for those with a low level of evidence. 

Together with an updated synthesis of available literature evidence, this report also includes guidance on practical considerations for the management of PD with safinamide. Concretely, the panel of experts suggests treatment approaches for managing dyskinesias or switching from rasagiline to safinamide. Moreover, the panellists also indicate a list of patient profiles that may benefit from safinamide therapy. In conclusion, this report provides updated expert consensus on the use of safinamide covering a wide range of clinical scenarios. 

## Figures and Tables

**Table 1:** Main efficacy and safety outcomes reported in pivotal trials of safinamide in Parkinson’s disease.

Study–Author	PD Stage	Sample Size	Design	Efficacy	Safety
Study 015 Stocchi et al. [11]	Early	270	Randomized, double-blind, placebo-controlled, parallel-group Treatment groups: Safinamide 200 mg; Safinamide 100 mg; Placebo.	UPDRS-III: significant differences between safinamide 100 mg/day vs placebo (−6.0 vs. −3.6; *P* = 0.0419), but not 200 mg/day vs. placebo.	Most common TEAEs: nausea, headache, abdominal pain (upper), vomiting, pyrexia, cough, hypertension, blurred vision, gastritis, peripheral edema, nasopharyngitis, dizziness, back pain, and tremor.
Study 017 Schapira et al. [12]	Early	227	Randomized, double-blind, placebo-controlled, parallel-group 12-month extension study of Study 015. Treatment groups: Safinamide 200 mg; Safinamide 100 mg; Placebo.	Difference in median time to intervention safinamide vs. placebo (93 days; *P* = 0.3342). Post-hoc analyses: Significantly higher changes in UPDRS-III in the safinamide 100 mg group versus placebo (*P* = 0.0264).	Most common TEAEs: back pain, scotoma, dizziness, blurred vision, upper abdominal pain, nausea, and hypertension (100 mg/day safinamide), cataract, upper abdominal pain, gastritis, and pain in extremity (200 mg/day safinamide).
Study 016 Borgohain et al. [6]	Mid-to-late	669	Randomized, double- blind, placebo-controlled, parallel-group. Treatment groups: Safinamide 100 mg; Safinamide 50 mg; Placebo.	UPDRS-III: mean difference vs. placebo: safinamide 50 mg/day −1.8, *P* = 0.0138, safinamide 100 mg/day −2.6, *P* = 0.0006). Increase in ON time without troublesome dyskinesia vs. placebo: 0.51 (50 mg/day) and 0.55 (100 mg/day) h. Change in OFF time vs. placebo: −0.6 (safinamide 50 and 100 mg/day). Comparable DRS between safinamide and placebo groups.	Most common TEAEs were nervous system disorders, general disorders and gastrointestinal disorders. Dyskinesia was more common in the safinamide group, worsening of PD and depression in the placebo group.
Study 018 Borgohain et al. [7]	Mid-to-late	544	Randomized, double-blind, placebo-controlled, parallel-group, 18-month extension to Study 016. Treatment groups: Safinamide 100 mg; Safinamide 50 mg; Placebo.	Long-term improvements in UPDRS part II, III, and IV total scores with safinamide 100 mg/day. Increase in ON time from baseline was 1.01 h in safinamide 50 mg/d group (*P* = 0.0031) and 1.18 h in the safinamide 100 mg/d group (*P* = 0.0002), compared with placebo (0.34 h). Reduction in DRS sores of 31%, 27%, and 3% in safinamide 50 mg/day, 100 mg/day, and placebo, respectively (*P* = NS).	Most common TEAEs (≥10 patients): cataract, asthenia, pyrexia, fall, back pain, dyskinesia, worsening of PD, headache, and insomnia. Incidence of new/worsening dyskinesia similar to placebo.
Study Settle Schapira et al. [8]	Mid-to-late	549	Randomized, placebo-controlled clinical trial. Treatment groups: Safinamide 50 mg → 100 mg; Placebo.	UPDRS-III: difference vs. placebo −1.82; *P* = 0.003). Increase in ON time without troublesome dyskinesia vs. placebo: 0.96 h, *P* < 0.001. Decrease in OFF time vs. placebo: 1.03 h, *P* < 0.001. Comparable DRS scores between safinamide and placebo groups.	The most common TEAEs were dyskinesia (14.6% safinamide group vs. 5.5% placebo group), falls (6.6% vs. 3.6%), urinary tract infections (6.2% vs. 4.4%), nausea (5.8% vs. 5.5%), and headache (4.4% vs. 6.2%). Dyskinesia was more frequent in the safinamide group vs. the placebo group. Severe dyskinesias: 1.8% in patients receiving safinamide and 0.4% receiving placebo.

UPDRS-III, Unified Parkinson’s Disease Rating Scale part III; TEAEs, treatment-emergent adverse event; DRS, Dyskinesia Rating Scale; PD, Parkinson Disease.
