# Erratum: Pagonabarraga, J.; et al. A Spanish Consensus on the Use of Safinamide for Parkinson’s Disease in Clinical Practice. *Brain Sci.* 2020, *10*, 176

**DOI:** 10.3390/brainsci10050313

**Published:** 2020-05-22

**Authors:** Javier Pagonabarraga, José Matías Arbelo, Francisco Grandas, Maria-Rosario Luquin, Pablo Martínez Martín, Maria Cruz Rodriguez-Oroz, Francesc Valldeoriola, Jaime Kulisevsky

**Affiliations:** 1 Movement Disorders Unit, Neurology Department, Hospital de la Santa Creu i Sant Pau, 08041 Barcelona, Spain; jkulisevsky@santpau.cat; 2 Department of Medicine, Autonomous University of Barcelona, 08193 Barcelona, Spain; 3 Centro de Investigación en Red sobre Enfermedades Neurodegenerativas (CIBERNED), 28031 Madrid, Spain; 4 Movement Disorders Unit, Neurology Department, Hospital Universitario San Roque, 35001 Las Palmas, Spain; jmarbelo@gmail.com; 5 Department of Medicine, Universidad Fernando Pessoa-Canarias, 35450 Las Palmas, Spain; 6 Movement Disorders Unit-CSUR, Neurology Department, Hospital General Universitario Gregorio Marañón, 28007 Madrid, Spain; francisco.grandas@salud.madrid.org; 7 Department of Medicine, Universidad Complutense de Madrid, 28040 Madrid, Spain; 8 Movement Disorders Unit, Clínica Universidad de Navarra (CUN), 31008 Pamplona, Spain; rluquin@unav.es; 9 Navarra Institute for Health Research (IdiSNA), 31008 Pamplona, Spain; 10 Instituto de Salud Carlos III, 28029 Madrid, Spain; pmm650@hotmail.com; 11 Centro de Investigación Biomédica en Red sobre Enfermedades Neurodegenerativas (CIBERNED), 28031 Madrid, Spain; 12 Neurology and Neuroscience Unit, Clínica Universidad de Navarra (CUN), 31008 Pamplona, Spain; mcroroz@unav.es; 13 Centre for Applied Medical Research (CIMA), 31008 Pamplona, Spain; 14 Neurosciences Institut, Hospital Clinic de Barcelona, 08036 Barcelona, Spain; FVALLDE@clinic.ub.es; 15 Department of Medicine, Universitat de Barcelona, 08036 Barcelona, Spain; 16 Biomedical Research Institute (IIB-Sant Pau), 08041 Barcelona, Spain; 17 Department of Medicine, Universitat Oberta de Catalunya, 08018 Barcelona, Spain

We would like to submit the following erratum to our recently published paper [1] due to the errors in the abstract. We request (1) the removal of the word “motor” from the first line of the abstract, so that the sentence reads: “Safinamide is an approved drug for the treatment of fluctuations in Parkinson’s disease (PD)”, and (2) that the word “OFF” is changed to “ON” in line 6 of the abstract, so that the sentence reads: “Safinamide significantly improves the mean daily ON time without troublesome dyskinesias.”

We apologize for any inconvenience caused to our readers.
